# DNA Origami Reorganizes upon Interaction with Graphite: Implications for High-Resolution DNA Directed Protein Patterning

**DOI:** 10.3390/nano6110196

**Published:** 2016-10-31

**Authors:** Masudur Rahman, David Neff, Nathaniel Green, Michael L. Norton

**Affiliations:** 1 Parabon Nanolabs, Huntington, WV 25701, USA; masud@parabon.com; 2 Molecular and Biological Imaging Center, Marshall University, Huntington, WV 25755, USA; dneff@marshall.edu; 3 Department of Natural Sciences, Northeastern State University, Broken Arrow, OK 74014, USA; green66@nsuok.edu; 4 Department of Chemistry, Marshall University, Huntington, WV 25755, USA

**Keywords:** DNA origami, graphene, graphite, atomic force microscopy, single-stranded DNA, double-stranded DNA, DNA -based nanostructures

## Abstract

Although there is a long history of the study of the interaction of DNA with carbon surfaces, limited information exists regarding the interaction of complex DNA-based nanostructures with the important material graphite, which is closely related to graphene. In view of the capacity of DNA to direct the assembly of proteins and optical and electronic nanoparticles, the potential for combining DNA-based materials with graphite, which is an ultra-flat, conductive carbon substrate, requires evaluation. A series of imaging studies utilizing Atomic Force Microscopy has been applied in order to provide a unified picture of this important interaction of structured DNA and graphite. For the test structure examined, we observe a rapid destabilization of the complex DNA origami structure, consistent with a strong interaction of single-stranded DNA with the carbon surface. This destabilizing interaction can be obscured by an intentional or unintentional primary intervening layer of single-stranded DNA. Because the interaction of origami with graphite is not completely dissociative, and because the frustrated, expanded structure is relatively stable over time in solution, it is demonstrated that organized structures of pairs of the model protein streptavidin can be produced on carbon surfaces using DNA origami as the directing material.

## 1. Introduction

The history of imaging of DNA on graphite substrates using probe microscopy is very contorted. Because it is ultra-flat and readily cleaved to provide clean, new surfaces and conducting, graphite was once considered an ideal substrate for DNA imaging studies using the first scanned probe technique, scanning tunneling microscopy, which requires a tunneling current for imaging. Around 1992, a combination of factors led to a precipitous decline in the funding of, and imaging studies of, DNA on graphite [1]. In particular, articles were published that cast doubt on the veracity of previously published DNA images obtained on graphite, indicating that graphite itself can mimic the structure of DNA [2,3], This, coupled with the rise of atomic force microscopy (AFM), an imaging tool free from the requirement of a conductive substrate, led to the overwhelming use of mica as a substrate for imaging DNA and DNA-based nanostructures when scanned probe approaches are employed. The current revolution in DNA-based structure fabrication and the rapid evolution of AFM capabilities, including the use of much more sensitive mechanisms for force sensing and higher resolution probes, argues for an evaluation of the potential of graphene as a useful substrate for the emerging DNA origami (DO) technology. While the focus of this paper is on graphite, the relationship with graphene, which may be considered the “top layer” of graphite, is certainly important. The precise placement of proteins, nanoparticles, and molecular species with nanoscale precision onto graphene or few layer graphite would enhance the rate of integration of carbon-based materials, as nano-optical or electronic components, into a wide variety of sensing and reporting (i.e., transducing) systems [4]. Thin, multi-layer graphene has exceptional features beyond its semi-metallic electronic properties. It displays remarkable mechanical strength, flexibility, and biocompatibility. Simultaneous with the rise in interest in carbon-based systems has been the development of the scaffolded form of self-assembled DNA. DNA origami is now a powerful tool to organize various molecules at the nanometer scale [5,6,7,8]. Numerous recent reports demonstrate the successful use of DO as a structural building block to make a variety of architectures ranging from simple periodic arrays to arrays with complex patterns addressable at multiple unique sites [8,9,10,11,12,13,14,15,16,17,18,19,20]. The development of methods to use self-assembled DNA to pattern species on unmodified graphite could significantly expand the range of applications for graphite in the realms of nanoelectronics, biosensors, and nano-optics. The interaction of DNA with this 2D form of carbon is less well studied than the interaction of DNA with the zipped-up form of graphite, single-walled carbon nanotubes (SWCNT). Single-stranded DNA (ssDNA) wraps helically on SWCNT and essentially forms a bridge between the hydrophobic CNT and aqueous media. The DNA presents its ionic backbone to solution, producing a hydrophilic coating, which leads to the use of DNA to aid in the effective dispersion of CNTs into the aqueous phase [21,22,23,24]. ssDNA offers the intriguing possibility of similar interaction with the two-dimensional carbon surface of graphite, based on an enhanced π–π stacking interaction between the surface and the planar aromatic nucleotide bases, which is augmented by the additional ionic contribution from the phosphate backbone [25,26,27,28]. The great majority of DNA in origami is usually double-stranded (dsDNA) by design, and only through a reorganization of its structure that would disrupt its intrinsic π–π stacking and hydrogen bonds could it interact through π–π stacking interactions with the graphite surface. This significant barrier results in an apparently limited binding to graphene and modified graphene, as reported in recent publications [26,29]. A major research objective is the generation of high-resolution patterns of species, controlled by the origami design, on the conductive, and extremely flat, carbon surface. In this approach, one could consider the carbon surface as acting as an electrical ground plane, while active components would be suspended at precise locations on the top of a DNA-based nanostructure. Our goal, therefore, is to develop a “molecular” lithography method, that is, a method for the patterned placement of single molecules of “soft materials” (proteins and organic molecules), which maintains the integrity and electronic properties of the carbon substrate surface and builds on top of it, in a bottom-up approach. DO is well known to maintain its high-resolution features on mica [30] and SiO_2_ [31]. Recently, Jin et al. [32] demonstrated that DNA nanostructures retained their designed structures when deposited on chemically treated graphene. Similarly, DO were observed to maintain their structures when adsorbed on graphene oxide (GO) [29]. A method that enables high-resolution patterning—through the intermediary of DNA nanostructures designed with precision—of species on the native carbon surface would be of value because it would avoid introducing randomly structured intervening molecules that could interfere with the electronic or optical coupling between the carbon and the species of interest. For soft molecules, truly molecular lithography, at nanometer resolution, requires the control only possible at this time through DNA-directed assembly [33].

In order to define and therefore enable us to address the challenges associated with implementing this precision lithography, which as its basis must entail binding origami to graphite, we explored the intrinsic binding of DNA origami to graphite, in the form of highly oriented pyrolytic graphite (HOPG). We describe and discuss structural observations, made using AFM, when the reaction between the HOPG surface and origami-containing solution is performed using the same conditions usually employed with the more common substrate mica. Because the HOPG surface is immediately passivated with single-stranded DNA in such a preparation, an alternative, “neat” preparation is demonstrated. A significant surface interaction, leading to the production of a remarkably different morphology for this test structure, is reported. Finally, because these disruptive effects are limited, we demonstrate that a designed pattern of pairs of the protein molecule streptavidin, and therefore presumably other materials, can be maintained through this structural reorganization.

## 2. Results

### 2.1. Contrasting DNA Origami Interactions with Mica and Graphite Substrates

#### 2.1.1. Test Object Deposition on Mica (Control)

**Figure 1:**
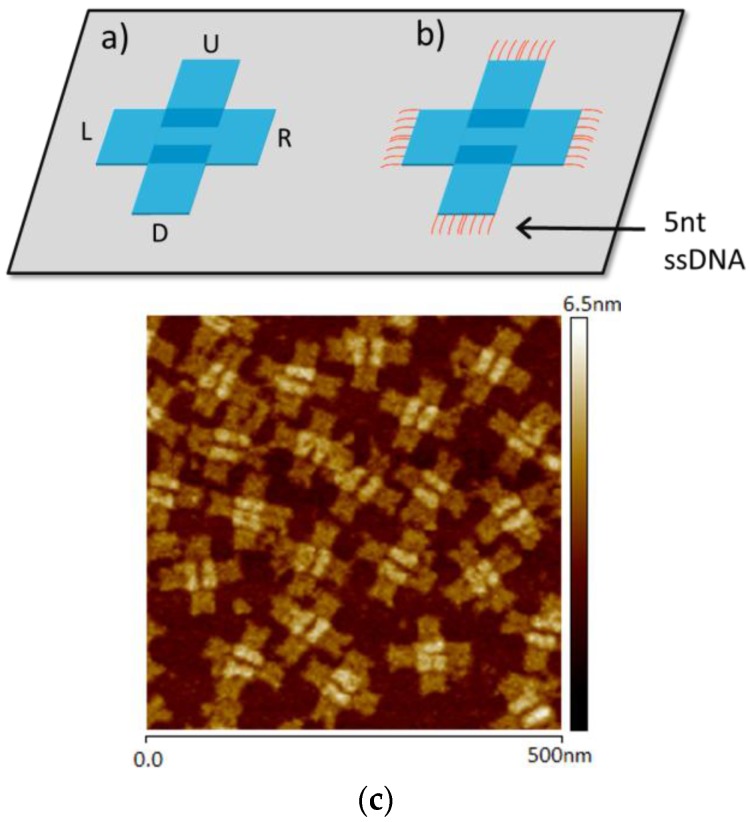
Schematic drawings of the cross-shaped DO with long (cDO) (**a**) and short (cDO_E_) (**b**) ssDNA terminations. Representative atomic force microscopy (AFM) image of cDO on mica (**c**).

For this study, we used cross-shaped DO (cDO). Two rectangular planks, one stacked on top of the other, are bound with perpendicular orientations to generate the cross-like origami structure designed by Liu [34]. The coordinate system and structure of two test structures are provided in Figure 1a,b. The cDO structure is composed of a completely base-paired core structure with each of the 12 helices contained in the end of each arm connected by a 32-base hairpin-like region of single-stranded scaffold. In the second test structure, all scaffold bases are paired, and there are five nucleotide ssDNA extensions projecting from the termini of each of the six helices in each of the four arms. Generation of these structures and their sequences are discussed in the Supplementary Materials (Table S1). The cross-shaped geometry was selected as the test structure for several reasons. The structures are highly reproducible experimentally and contain “landmarks” or high points that are easily observable. An AFM image of the cDO, which has dimensions of ~100 nm × 100 nm and 2.8 nm high “stripes,” is shown in Figure 1c. The orthogonal cross-shape structure is extensible by design, readily forming two-dimensional [34] and one-dimensional [35] arrays.

#### 2.1.2. Adsorption of DNA Origami from Solutions Containing Excess Staples onto HOPG

**Figure 2:**
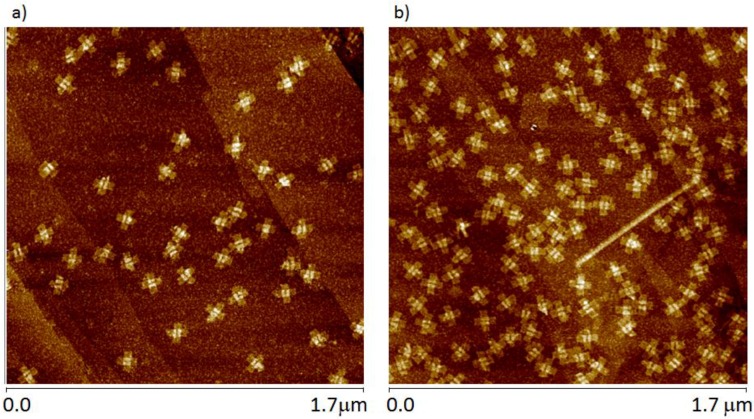
AFM images of the cDO (with long ssDNA segments) (**a**) and cDO_E_ (with short 5-base ssDNA extensions) (**b**) on highly oriented pyrolytic graphite (HOPG).

Historically, DNA origami is usually produced at plasmid concentrations between 1 and 10 nM and individual staples between 5× and 100× the plasmid concentration. Because there are also on the order of 200 different staples per construct, the solution concentration of single-stranded DNA can range from 1 to 200 μM. In the case of preparing samples for AFM imaging using mica substrates, samples can be prepared by directly dosing the surface with solutions containing this mixture: nanomolar concentrations of completely formed origami and very high concentrations of single-stranded DNA. When this approach to sample preparation is used in the case of HOPG substrates, the apparent product is a low-density population of the surface with well-defined origami. A representative image displaying this sparse coverage is provided in Figure 2. For similar dosing times, the surface occupation is higher for the constructs containing short single-stranded terminations at the end of each arm. The morphology of the immobilized structures appears to be identical in both cDO and cDO_E_. 

The image in Figure 2a might mistakenly be interpreted to mean that DNA origami, because it is mostly double-stranded and in this double-stranded form is hydrophilic, and because the planar bases are internal and therefore not exposed, lacks a mechanism to bind with the hydrophobic graphite surface, yielding low coverage. This is not, however, the case. Our previous studies and those of Husale et al. have demonstrated that ssDNA, including that from excess staple strands in a non-purified origami mixture, binds to the graphite/graphene surface and forms a 1–1.5 nm thick layer [26,36].

**Figure 3:**
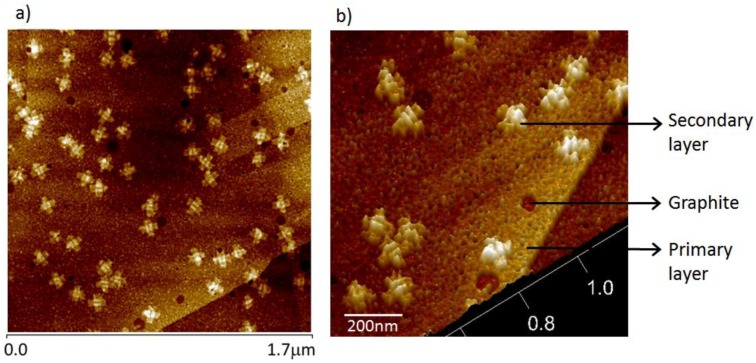
AFM images of cross-shaped origami deposited from a solution containing staples. Well-defined origami are visible on top of a primary layer of single-stranded DNA. Large pores are visible in the low magnification top projection image (**a**); smaller pores in this layer are visible in the 3D rendering of the bottom center portion of image (**a**) provided in image (**b**).

Indeed, closer inspection of the AFM images of origami samples prepared from solutions containing excess staples reveals that there is actually a relatively dense amorphous sub-layer underneath, and supporting, the visible origami structures (see Figure 3). Evidence for this underlayer is two-fold. First, the HOPG surface roughness is significantly greater than the intrinsic carbon surface roughness. Secondly, and more compelling, is the appearance of holes or deep (1–1.5 nm) pores in the layer that reach down to the graphite surface. This apparent monolayer coverage is consistent with an excellent study that revealed the growth of a dense, porous mat structure, resulting from dosing the HOPG surface with single-stranded DNA homopolymers that were 10 mers in length [37]. 

The size and landmark definition of the observed origami structures closely matches the appearance of these origami on mica. This observation might lead one to believe that the graphite surface is a suitable direct replacement for mica for origami imaging. The surface coverage is lower than that observed on mica for comparable dosing times, which could lead one to conclude that it is the graphite surface, rather than the monolayer of ssDNA on the surface, that inhibits DO binding. In our experience, the observation of origami binding on top of another origami on mica is quite rare, and this rarity is ascribed to electrostatic DNA–DNA repulsions. It is therefore unexpected that the origami constructs observed in Figure 3, which are on top of an ssDNA layer, are so plentiful. Our suggested explanation for this binding of DO constructs on this disorganized DNA monolayer involves entanglement (random base pairing) between the disordered monolayer constituents and strands composing the origami construct or interaction of single-stranded components of the origami with sparse regions of free graphite surface. The observed higher density of DO binding to the monolayer surface when the DO construct is equipped with shorter regions of unpaired bases (compare Figure 2a with Figure 2b) appears paradoxical, but may be explained by comparing the relative entropy changes associated with binding for these two constructs. It is important to note that the porosity of this monolayer is quite apparent in samples imaged in air after drying and almost invisible, in our experience, when AFM images are acquired under a buffer solution as demonstrated in Figure S1. Potential reasons for this difference in visibility in the two environments include that large pore formation may be an artifact, developing during the dehydration process, or that there is sufficient surface mobility, under solution conditions, for the monolayer to spread more evenly, perhaps under the influence of the AFM tip.

From these observations, it is clear that if one is to study the origami/surface interaction on HOPG, it is necessary to filter out the excess staples from the DO solution before the deposition reaction is performed. Due to the strong interaction between ssDNA and graphite, this procedure is required in order to avoid formation of the spontaneous, intervening ssDNA monolayer on the graphite surface.

#### 2.1.3. Adsorption of Purified DNA Origami onto HOPG 

**Figure 4:**
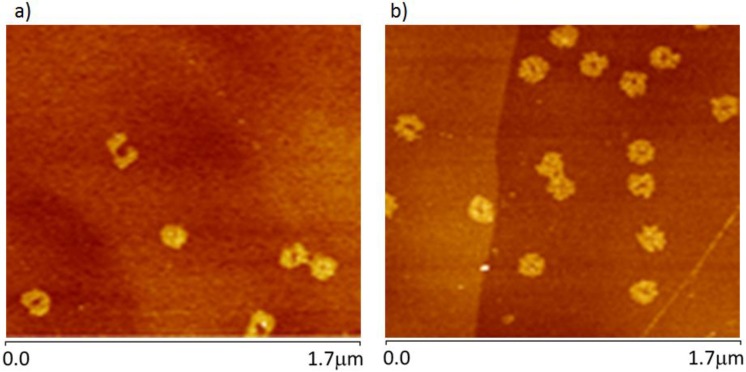
AFM topography images of cDO reacted with HOPG for 10 seconds: (**a**) (cDO); (**b**) constructs with short ssDNA extensions (cDO_E_).

Based on the observation that the aromatic bases of ssDNA help wrap DNA around the CNT surface via π–π bonding [22,24,38], it was expected that the introduction of short linear single-stranded regions at the end of each arm would assist in rapidly binding origami to the graphite surface. The effect of eliminating interfering excess single-stranded DNA from the deposition solution, coupled with the introduction of short single-stranded regions into the structure, is apparent in the images shown in Figure 4, which are discussed below. 

When such purified (method discussed below) solutions of cDO and cDO_E_ are used, the short ssDNA extensions clearly enhance the rate of binding of dsDNA origami onto the graphite substrate. We have evaluated the bound surface density of purified cDO (Figure 4a) and cDOa (Figure 4b) on HOPG 10 s after deposition of a 0.3 nM solution. Imaging reveals that ~10 times more cDO_E_ (~37 DO/micron^2^) as compared with cDO (~4 DO/micron^2^) are bound to the graphite surface.

The differential rates of deposition onto HOPG of the constructs containing short single-stranded regions are again higher than those observed for a system with longer, hairpin-shaped single-stranded regions. In addition, a remarkable change in origami morphology is also observed in these excess staple-free experiments. 

**Figure 5:**
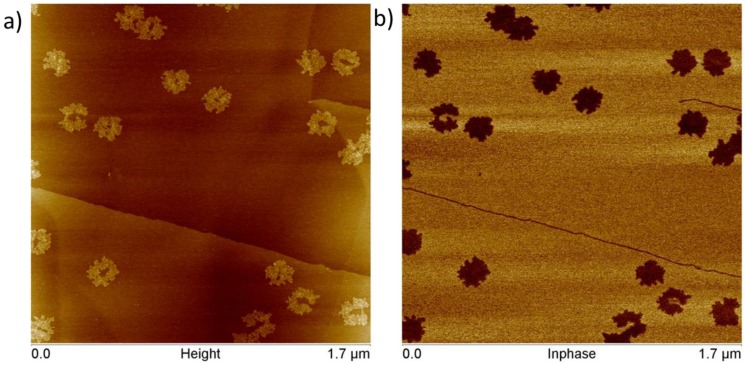
Simultaneously acquired AFM topography (**a**) and in-phase (**b**) images of cDO_E_ deposited onto HOPG.

When centrifuge-filtered origami solutions are used (solutions containing no additional ssDNA), that is, when deposited directly on graphite, the DO do not maintain the same sharply defined form one observes when they are deposited on mica. Instead, they appear to partially disintegrate upon interacting with graphite, in a reconstructive process which reduces their topographic contrast and expands their footprint on the HOPG surface. As can be seen in Figure 5a, the constructs become more circular in appearance, and the contrast due to the designed raised markings at the center of the constructs has become unobservable, changing from 2.8 nm to 0.85 nm, much less than the value observed for the designed double-thick layer of double-stranded DNA on mica (analysis and data in Figure S2 and Tables S2 and S3. We conclude that this is consistent with conversion to surface-sorbed ssDNA. This morphological change alone suggests that the nature of the substrate interaction has changed significantly with respect to the ssDNA adsorbate layer case discussed above, and that the origami are now truly bound to the graphite surface. An AFM phase image is provided in Figure 5b as a reference, since PEAKFORCE (Bruker AFM mode) is differentially sensitive to the properties of the materials under the tip.

**Figure 6:**
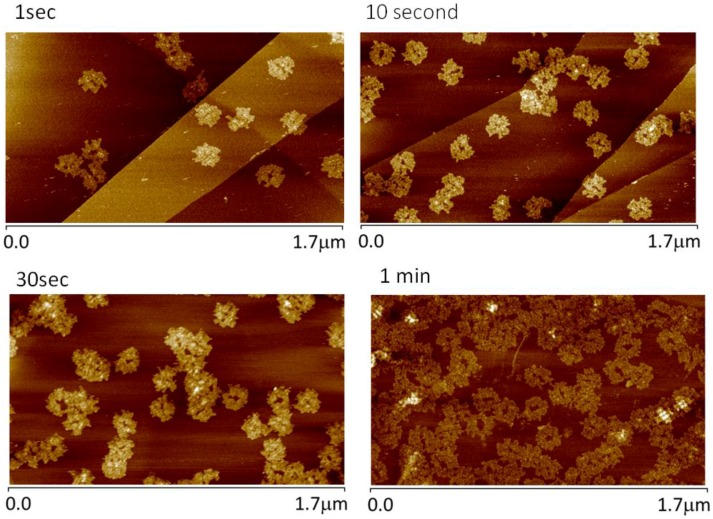
AFM images of different HOPG surfaces exposed to purified, 0.3 nM cDO_E_ solution for periods ranging from 1 s to 1 min.

Through imaging HOPG surfaces that were exposed to 0.3 nM dialyzed origami solutions for periods of 1, 10, 30, and 60 s and then quenched via rinsing and drying, we observed that DO adsorption on graphite results in an almost immediate change in morphology that does not appear to evolve over time. Example images corresponding to these time points are provided in Figure 6.

Although DNA origami are not single molecules in the formal sense, our experiments show that purified DO, even when partially reconstructed through interaction with HOPG, maintain discrete boundaries and are recognizable as individual origami. Interestingly, there seems to be no trend in increasing boundary extent with increasing exposure time to HOPG. Non-passivated graphite apparently disrupts the bonding between the ssDNA staples and the ssM13 scaffold of the origami until the boundaries of each DO expand as ssDNA staples/ssM13 bind via *pi*-stacking to the HOPG surface. Once the bases of DO staples are π-stacked with the HOPG, the structures no longer appear to expand across the HOPG surface. The perimeter of a single adsorbed cDO_E_ (average of all time points) surrounds an area of ~13,000 nm^2^. A central void (bare HOPG), apparent within this perimeter, accounts for ~1500 nm^2^ of this total footprint.

The single-stranded nature of the bound origami on these surfaces is supported by the observation that the AFM-determined line profiles indicate that the height of cDO_E_ on HOPG is ~0.85 nm, which is much less than the usual dsDNA thickness measured at the arms of the structure, on mica, of ~1.4 nm (analysis and data provided in Tables S2 and S3).

With longer deposition times, more origami bind to the graphite substrate (Figure 6). At these higher surface coverages, adjacent origami appear to lose their defined boundaries at their points of contact. This intergrowth is best observed at the one-minute time point shown in Figure 6, where packing makes the individual origami unrecognizable. However, those separated individuals that were already fully bound onto HOPG do not appear to change significantly in the extent of their coverage (footprint) as a function of time. Also observable at the one-minute time point is the beginning of the formation of a second discrete layer of well-defined origami depositing on top of the primary layer. The primary layer, composed of “expanded” origami, and the secondary layer can be clearly differentiated (white, well-formed origami) after one minute of deposition. It would again appear that DNA adsorption on top of DNA, quite uncommon in the case of origami deposited on mica, is observed due to either the presence of unpaired bases associated with single-stranded DNA on graphite (in this case, the unpaired bases would be bases that are components of the first, “expanded” origami layer, which are not bound to the surface via π–π bonding) or the origami in the secondary layer having only limited contact with the graphene substrate, sufficient to enable immobilization but not enough to cause significant bond re-organization.

In solution phase studies, we have not observed origami dissociating from the HOPG surface. It would therefore appear that the binding of origami onto HOPG is practically irreversible. The reorganization of the cDO_E_ even at very short deposition times indicates a coupled binding-reorganization process. Previous molecular dynamic simulations have shown that ssDNA placed on graphene adopts a conformation in which bases alternate from one side to the other side of the phosphate backbone [22,26,39,40]. Once cDO_E_ contacts graphite, it appears to flatten immediately and partially lose its structural definition; this structural change appears to be finite but unavoidable. In order to further partially characterize this process, and in order to evaluate the potential use of origami to organize proteins on a graphite surface, we deposited origami containing pairs of streptavidin molecules, positioned via biotin-labeled staples, onto the HOPG surface. 

### 2.2. Protein Patterns on DNA Origami Are Perturbed during Adsorption onto Graphite Substrates

**Figure 7:**
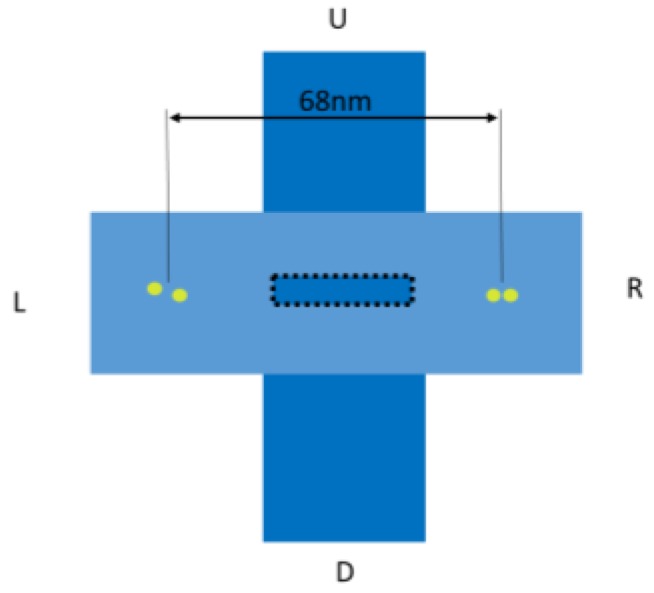
Schematized streptavidin-labeled origami construct.

The above studies indicate that the cross shaped origami undergoes great structural reorganization upon interaction with the graphite surface. The staples in DNA origami can serve two purposes. Their main role is to direct and maintain the designed structure, but because these DNA strands can be modified, they can also be used to direct the localization of a variety of species by binding them to the surface of the origami. Because the staples cannot usually be identified as independently recognizable species in AFM images of origami, we chose to label a small subset of the staple strands with the protein streptavidin. Acting as stable and readily recognizable topographic markers, a pattern of two streptavidin protein molecules, shown schematically in Figure S3, would be used to enable determination of the location of these strands after the reorganization. For this study, four of the component staples of the cross-shaped DO were modified with biotin, two staple strands on each of two opposing arms (left and right arms as defined in Figure 7).

**Figure 8:**
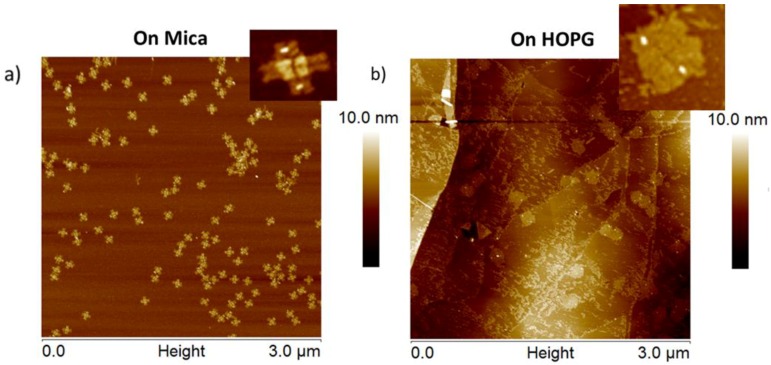
AFM images of cDO modified with streptavidin, on (**a**) mica and on (**b**) HOPG.

After annealing, the pre-formed origami was incubated with streptavidin to yield >85% site modified origami tiles, providing ready topographic markers for protein, and therefore staple, localization. The origami were purified to remove excess staples and streptavidin, then deposited on mica and on HOPG. A representative image of these origami, deposited on mica, is presented in Figure 8a. The appearance of these well-defined origami structures (high contrast, compact arms, and landmark topographic features) is highly characteristic of deposition on mica. 

Our previous deposition time course study demonstrated that 10 s exposure was sufficient to provide useful numbers of consistent cDO shapes on HOPG without significant surface crowding. This deposition time was therefore chosen for the protein patterned origami deposition studies described here. An AFM image of streptavidin-modified origami deposited on the HOPG surface is presented in Figure 8b. Two noteworthy features are observed. First, the streptavidin markers are significantly displaced from both the designed separation and even from their anticipated locations in the expanded structure. Whereas the designed separation distance between sites for the native structures was 68 nm (73 nm observed, see Table S5), the reorganized structures yield an average separation of 107 nm (data in Table S5). If the structure had simply expanded linearly, an expanded separation of ~90 nm would have been anticipated. Secondly, the fraction of doubly labeled origami (streptavidin on each of the arms) is much less than >85% occupancy observed for the input materials (Figure S4). Because the “new” labeled staple locations are very near the edge of the expanded origami, it is quite possible that staples near the edges migrated further on the surface than those more central to the construct, which may have their motion impeded by nearby staples. It may also be noted that, in the case of the re-organized, expanded systems, in several instances two streptavidin molecules are observed with small separations (~30 nm). Because the design of the unexpanded, native system contains pairs of staples labeled with biotin in very close proximity (designed separation of 5 nm), two separate, readily discernable streptavidin molecules are not observed in images of these compact structures on mica. Clearly such double occupancy exists, and is made visible by the reorganization that expands the origami structure. These observations are consistent with significant loss of staple-scaffold binding accompanying the adsorption event.

## 3. Discussion

A novel effect accompanying origami immobilization on graphite has been observed. This effect may not have been previously observed due to the fact that excess single-stranded DNA in solution can mask the interaction by passivating the surface. The observed effect, a restructuring of the origami, may be attributed to the strong interaction between single-stranded DNA and π-conjugated carbon surfaces, an interaction that apparently readily competes with the hydrogen bond interactions in double-stranded DNA. This restructuring is reflected in a significant expansion of the “footprint” of the origami and a concomitant decrease in the height of the constructs to levels significantly below values observed, on mica substrates, for origami sections presumably composed of dsDNA. Similar results are anticipated for the related material graphene.

The test structure studied, a cross-shaped origami, is significantly restructured upon binding to the graphite surface. Signatures of this restructuring include a loss of sharply defined structural features, a significant change in height, and an expansion of substrate area covered by a factor of two. Although AFM, in our hands, is not able to resolve the structure of the reorganized DNA assembly, the loss of structural integrity would seem to indicate a loss of the ability of the staple species to direct the organization of the structure. If the staple/scaffold hydrogen bonding interaction is disrupted and replaced with substrate–DNA base interactions, then not only would the structure be required to spread in area, to maximize this contact, but also the height of the structure should transition from that corresponding to double-stranded DNA to a height appropriate for single-stranded DNA. 

An almost complete transformation to a single-stranded state is consistent with the changes observed in the structures reported here. It may even be speculated that the early failures to image double-stranded DNA using scanning tunneling microscopy (STM) on graphite may have been related to this apparent melting phenomenon. Crossover points present in the design of the origami may generate points of frustration, limiting surface diffusion in this spreading process. The presence of larger quantities of ssDNA at the designed edges of these DO structures may also “pin” the periphery of the structures, limiting this spreading. However, significant differences in spreading for the two models structures were not observed in these studies. It does appear that the rate of adsorption of origami to graphite can be impacted by the nature of the single-stranded component of these origami constructs. Short ssDNA extension strands, so called “sticky ends” at the edges of the origami construct produce a higher rate of origami binding to the graphite surface than longer hairpin loop-shaped strands, which have much more structural flexibility.

The transition to a single-stranded state could also result in at least partial loss of registration of the staples with the scaffold. In the study reported here, select staples were used to localize the protein streptavidin at two well-defined locations in the intact constructs. When imaged on mica, structures consistent with the designed, double-stranded DNA scaffold were observed. While the positions of these proteins, and presumably their associated staples, were significantly modified in the course of adhesion-associated restructuring and expansion, they remained localized on opposite sides of the resulting structures. This is interpreted to mean that at least some of the staples remain spatially localized, their surface diffusion frustrated by the intrinsic crossover structures of the origami, and therefore their locations are directed by the scaffold to remain within the footprint of the expanded origami. This maintenance of the protein pair or “dimer” structure implies that other proteins and particle types can be similarly organized and localized on the graphite surface, directed by DNA structures. The implications for the patterning of proteins and lithography on graphite using DNA as an organizing agent are significant. That this intrinsic reorganization occurs can be expected to lead to the development and use of new structural designs. For protein organization, such designs must either minimize the effects of adsorption or simply incorporate the expansion effects into designs for multicomponent surface structures. For lithography, there is the possibility that these expanded structures will be of greater value if they can be designed for close surface packing, because origami, due to the necessary crossovers, is essentially porous. 

The combination of the conductive ultraflat substrate graphite with the surface organizing capabilities of self-assembled scaffolded DNA structures to generate stable protein patterns has been demonstrated. This new combination represents an important advance because it can be anticipated to lead to the development of new nanosensing modalities that could not be implemented on insulating mica, the dominant current substrate for DNA-based structures.

## 4. Materials and Methods

**Cross-shaped DO:** The cross-shaped DNA Origami (CO) were prepared using methods published by Liu et al. [34]. Staples for particular modifications are described in the Supplementary Materials. Briefly, the mixture of staple strand DNA oligomers (IDT) 10 nM (including modified staple listing provided in Tables S1 and S4) and M13mp18 DNA genome (1 nM) was brought to a volume of 60 μL using 1× TAE buffer solution containing 40 mM Tris-HCl, pH 8.0, 20 mM acetic acid, 2.5 mM EDTA, and 12.5 mM magnesium acetate. Constructs were annealed in a slow cooling process, dropping the temperature linearly from 90 to 20 °C over a 13-h period. Streptavidin labeling was performed using a 20× excess of streptavidin (Fisher Scientific) incubated for 12 h. Excess staples and streptavidin were removed from the solution using the following purification method.

**Streptavidin (SA)-labeled origami preparation:** Freshly prepared 10 nM DNA origami solutions were incubated with 200 nM of SA (Thermo Fisher; Cat: 21,125, Waltham, MA, USA) for three hours at room temperature. 

**Purification of DNA origami and SA-labeled DNA origami:** To remove excess staples, anchor strands, and SA (from SA–origami solution), the origami solutions were filtered using 100 kDa MW centrifuge filters (Millipore YM-100, Darmstadt, Germany). Origami solutions were washed six times with 400 µL of 1× DO buffer using a bench top micro-centrifuge (Quikspin, Phenix Research Inc., Asheville, NC, USA). After filtration, the origami solutions were stored at 4 °C.

**HOPG**: HOPG substrates were purchased from Bruker Inc. (Billerica, MA, USA). Clean surfaces were generated by cleaving off surface layers using the scotch-tape method. 

**DO deposition**: 20 μL of freshly prepared (synthesis solution or as purified) DO solution were deposited on HOPG substrates and after selected times the substrates were rapidly rinsed (washed) with 0.5 mL distilled H_2_O and dried immediately with a dry nitrogen stream.

**AFM imaging**: All of the AFM imaging studies were performed using a Bruker Multimode 8 and Nanoscope VI controller (Billerica, MA, USA) in the SCANASYST-AIR mode or SCANASYST-FLUID mode, which uses PEAKFORCE tapping, a non-resonant, 2000 Hz tapping-like imaging mode. Air cantilevers are of the double-arm type, made of silicon nitride with a nominal spring constant of 0.4 N/m. Tips are made of silicon and have a nominal radius of 2 nm. Liquid cantilevers are of the double-arm type and are made of silicon nitride with a nominal spring constant of 0.7 N/m. Tips are made of silicon nitride and have a nominal radius of 20 nm. Unless otherwise stated, images were acquired in air.

## Supplementary Materials

The following are available online at http://www.mdpi.com/2079-4991/6/11/196/s1.
