# Supplementary material for: DNA Origami Reorganizes upon Interaction with Graphite: Implications for High-Resolution DNA Directed Protein Patterning
Source: Nanomaterials (Basel). 2016 Oct 31;6(11):196. doi: 10.3390/nano6110196 (PMC5245763; doi:10.3390/nano6110196)
Supplement: Supplementary file 1 [file nanomaterials-06-00196-s001.pdf]

Supplementary Materials: DNA Origami Reorganizes upon Interaction with Graphite: Implications for High-Resolution DNA Directed Protein Patterning

Masudur Rahman, David Neff, Nathaniel Green, and Michael L. Norton

1. cDO and cDO$_E$ Designs

The unmodified cross origami structure is generated by eliminating the staples in the list below from the Tile A design in the Liu et al. paper [1].

The cDO$_E$ (cross origami with extended staples) is identical to the Tile A design of Liu. That is, the staples with the 5-bp extensions as listed in Table S1 were used to make the CO$_E$ origami.

Table S1. List of staples deleted to generate cDO (non-extended staple origami).

CO-A-L1	TCCTGAACAAGAAAAAATCAACAATAGATAAGAGCAT
CO-A-L2	TTGCACCCAGCTACAAAAGATTAGTTGCTATTGCAAA
CO-A-L3	ATCCTAATAATAAGAGCAAGAGAATTGAGTTAAGCCCTATGG
CO-A-L4	GTCTTGTTTGAGGGGACGACGAACCGTGCATCTGCCAAAGGT
CO-A-L5	CGAATCCCGGGTACCGAGGTCTCGACTCTAGAGGATC
CO-A-L6	CTGTTAGCTGATTGCCCTTCACAGTGAGACGGGCAAC
CO-A-R1	CTGTTGTTAAATAAGAATAAAGTGTGATAAATAAGGC
CO-A-R2	CGAATAAATCGTCGCTATTAAATAACCTTGCTTCTGT
CO-A-R3	GTCTTAAATAAAGAAATTGCGTTAGCACGTAAAACAGAAGGT
CO-A-R4	ATCCTTATTCCTGATTATCAGAGCGGAATTATCATCATATGG
CO-A-R5	TGCTGAACCTCAAATAATCTAAAGCATCACCTGCAAA
CO-A-R6	ACATTGGCAGATTCACCTGAAATGGATTATTTAGCAT
CO-A-U1	AATAAGTTTATTTTGTCGCAAAGACACCACGGAGTGT
CO-A-U2	TGTAGCGCGTTTTCATGCCTTTAGCGTCAGACGTTCA
CO-A-U3	TGAGTAATTTACCGTTCCAGTGAAAGCGCAGTCTCTGTCTAC
CO-A-U4	CTATCGGTTTAGTACCGCCACATCACCGTACTCAGGAACTTG
CO-A-U5	GACATACTAAAGGAATTGCGAAGAATAGAAAGGAACA
CO-A-U6	CGTAAGAGGACTAAAGACTTTCGGCTACAGAGGCTTT
CO-A-D1	CGTAACGTTAATATTTTGTTAATATTTAAATTGTAAA
CO-A-D2	GACATTGAGTAATGTGTAGGTTTTTAAATGCAATGCC
CO-A-D3	CTATCATTAGATACATTTCGCTAGATTTAGTTTGACCACTTG
CO-A-D4	TGAGTATCAAAAAGATTAAGAAAGCAAAGCGGATTGCTCTAC
CO-A-D5	ATAACGCCAAAAGGAACAACTAATGCAGATACGTTCA
CO-A-D6	GGATATTCATTACCCAATCTTCGACAAGAACCAGTGT

2. Example of Difference Observed in ssDNA Background Imaging in Solution vs. Air (dry)

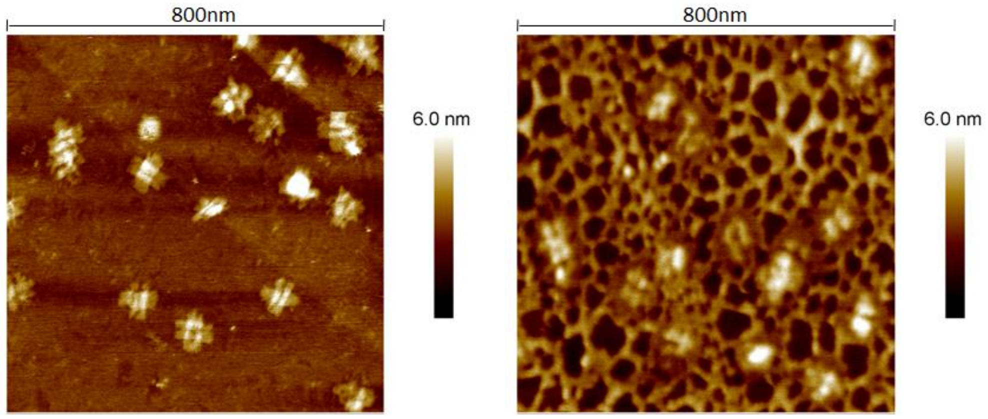

(**a**) AFM in buffer (**b**) AFM in air

Figure S1. Atomic force microscopy (AFM) images acquired in solution (**a**) and in air (**b**). Defects in the passivating layer (dark spots in image b) are apparent in images acquired in air.

The images above are of DNA origami, both on the same HOPG surface (although very close, these are not the same 800 nm × 800 nm regions). Figure S1a presents an image of DNA origami on HOPG imaged in liquid. This particular image was taken after HOPG had been exposed to a 0.3 nM origami solution for more than 50 min of continual scanning in a buffer (1× TAE w/12.5 mM $MgCl_2$). Figure S1b presents the same HOPG surface imaged in air after rinsing and drying. The passivating layer of DNA on the HOPG surface is hardly visible in the liquid scan but serves to protect the DNA origami from disintegration; this layer becomes quite obvious in the dry scan as a network of dried DNA (right).

3. Comparative Analysis of Height Data for cDO on Mica and Graphite Substrates.

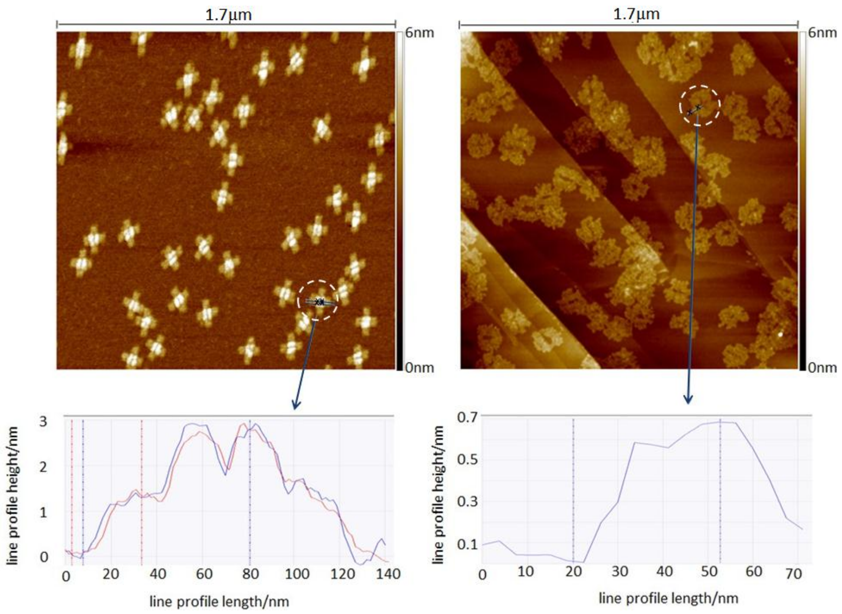

Figure S2. AFM images (imaged in air) of origami taken on mica (top left) and on graphite (top right). Example line profile of one origami construct appearing in the image on mica indicates the height, with respect to the surface of mica, of a single layer of dsDNA (the arm of the origami), and double layers of dsDNA (higher points). The line profile of HOPG (lower right panel) indicates a relatively flat profile for origami on graphite. Heights were measured as shown in example images with profile plots. Twenty-five measurements were taken such that the averages and standard deviations shown in Table S2 reflect the magnitude and variability of the height of DNA as measured in air by AFM in Peak Force Tapping feedback mode.

Table S2. Summary of observed DNA heights on mica and HOPG.

Parameter	dsDNA on mica	2× Deep dsDNA on mica	Difference dsDNA 2× Deep Minus dsDNA	ssDNA on HOPG
Average (nm)	1.43	2.82	1.39	0.85
SD (nm)	0.12	0.18	0.19	0.12

The DNA origami shown on the left was deposited on mica from a solution that was depleted of surplus staples (diafiltered) and applied to mica. The DNA origami on the right was depleted of surplus staples (diafiltered), and applied to HOPG (0.3 nM origami solution) for 5 min. Both surfaces were rinsed and blown dry with inert gas.

Data used to produce summary Table S2 above:

Table S3. Tabulated height data for DNA on mica and HOPG.

Parameter	dsDNA on mica	2× dsDNA on mica	difference 2× dsDNA minus dsDNA	ssDNA on HOPG
	1.3	2.7	1.4	0.69
	1.5	2.8	1.3	0.79
	1.3	2.4	1.1	0.9
	1.4	2.7	1.3	0.83
	1.4	3.3	1.9	0.75
	1.5	2.7	1.2	0.91
	1.3	2.8	1.5	1
	1.6	2.8	1.2	0.97
	1.5	2.7	1.2	0.78
	1.4	3.1	1.7	0.95
	1.3	2.6	1.3	0.96
	1.5	2.9	1.4	0.91
	1.4	2.9	1.5	1.2
	1.6	2.8	1.2	0.78
	1.6	2.9	1.3	0.85
	1.3	2.8	1.5	0.83
	1.4	3.1	1.7	0.81
	1.3	2.8	1.5	0.77
	1.4	2.7	1.3	0.83
	1.5	2.8	1.3	0.76
	1.6	2.9	1.3	0.71
	1.3	2.7	1.4	0.74
	1.6	2.9	1.3	0.63
	1.3	2.8	1.5	0.98
Average (nm)	1.43	2.82	1.39	0.85
SD (nm)	0.12	0.18	0.19	0.12

4. CO$_E$ Modified with Biotinylated Staples and Reaction with Streptavidin

In order to produce origami with a high probability of having at least one streptavidin label on each arm, an approach was employed that used two biotinylated staples per arm, providing a topographic signature for at least one staple per arm of the origami construct, as schematized in Figure S3.

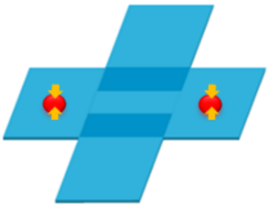

Figure S3. Schematized streptavidin labeled origami construct (yellow arrows = biotinylated sequences; red spheres = streptavidin).

The following staples of the Tile A design of Liu were replaced with biotinylated staples:

Table S4. Staple replacement list for biotinylated origami.

CO-M-9-BIOTIN	5′- GTG CCA AGG AAG ATC GAC ATC CAG ATA GGT T/3BioTEG/ -3′
CO-M-16-BIOTIN	5′- TAA GAA AAG ATT GAC CGT AAT GGG CCA GCT T/3BioTEG/ -3′
CO-M-74-BIOTIN	5′- AGT AGA AAA GTT TGA GTA ACA /3BioTEG/ -3′
CO-M-81-BIOTIN	5′- ATT GAA CCA ATA TAA TCC TGA TTG TCA TTT TG/3Bio/

5. Analysis of Streptavidin on Mica and on HOPG

Enlarged Images:

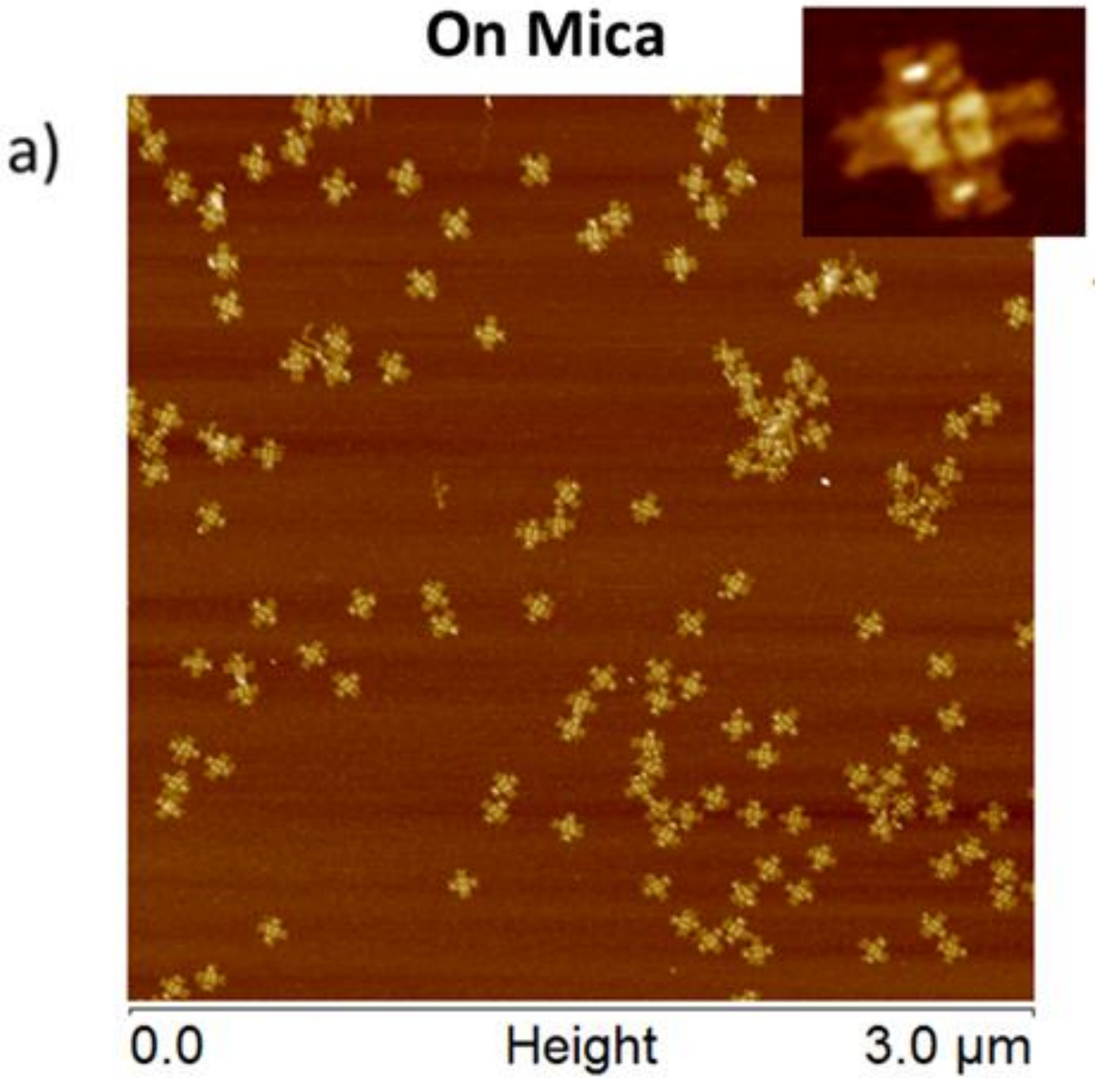

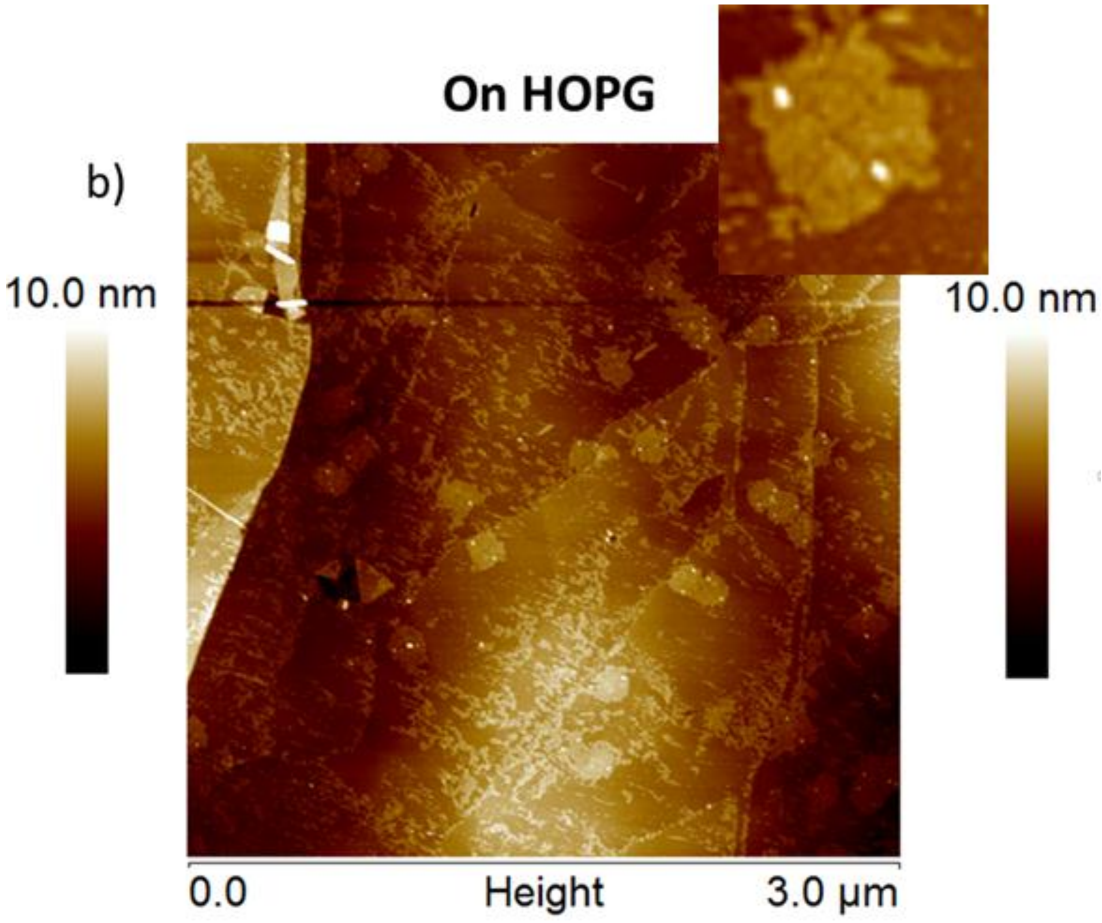

Figure S4. AFM image of streptavidin modified origami on mica (**a**) and on HOPG (**b**)

Measurements of distances between streptavidin molecules, on origami, in microns:

Table S5. Comparison of distances between streptavidin modifications

Streptavidin Separations	HOPG	Mica
	0.086	0.076
	0.113	0.066
	0.104	0.07
	0.102	0.069
	0.153	0.073
	0.108	0.073
	0.098	0.07
	0.089	0.077
	0.104	0.075
	0.096	0.078
	0.11	0.071
	0.106	0.076
n = 13	0.119	0.078
Average	0.107	0.073
SD	0.017	0.004

The yield of double-occupied origami (two streptavidin molecules, one on each of two opposing arms) is 88% for the "as-formed" (imaged on mica) (80 pairs of 90 origami observed) and 42% on graphite (13 with pairs of a total of 31 recognized origami on graphite).

Reference

1. Liu, W.; Zhong, H.; Wang, R.; Seeman, N.C. Crystalline two-dimensional DNA-origami arrays. *Angew. Chem. Int. Ed. Engl.* **2011**, *50*, 264–267.

© 2016 by the authors. Submitted for possible open access publication under the terms and conditions of the Creative Commons Attribution (CC-BY) license (http://creativecommons.org/licenses/by/4.0/).
